# Positioning a Paediatric Compounded Non-Sterile Product Electronic Repository (pCNPeRx) within the Health Information Technology Infrastructure

**DOI:** 10.3390/pharmacy4010002

**Published:** 2015-12-24

**Authors:** Richard H. Parrish

**Affiliations:** Meds4Kids Research Collaborative, Ltd, Edmonton, AB T6M 2J9, Canada; rhparrish2@yahoo.com; Tel.: +1-780-705-6857

**Keywords:** electronic prescriptions, health information technology, paediatric safety, drug standards, drug compounding, medical informatics application

## Abstract

Numerous gaps in the current medication use system impede complete transmission of electronically identifiable and standardized extemporaneous formulations as well as a uniform approach to medication therapy management (MTM) for paediatric patients. The Pharmacy Health Information Technology Collaborative (Pharmacy HIT) identified six components that may have direct importance for pharmacy related to medication use in children. This paper will discuss key positions within the information technology infrastructure (HIT) where an electronic repository for the medication management of paediatric patients’ compounded non-sterile products (pCNP) and care provision could be housed optimally to facilitate and maintain transmission of e-prescriptions (eRx) from initiation to fulfillment. Further, the paper will propose key placement requirements to provide for maximal interoperability of electronic medication management systems to minimize disruptions across the continuum of care.

## 1. Introduction

Often, paediatric patients receive medication therapy with products that are not commercially available until pharmacists prepare these products extemporaneously for dispensing [1]. The United States Pharmacopeia (USP) refers to these products as compounded non-sterile products (CNPs) [2]. As noted in other papers of this Special Edition, numerous gaps in the current medication use system, which includes these products, impede the complete transmission of electronically-identifiable and standardized formulations [3], and a uniform approach to medication therapy management services (MTM) for paediatric patients [4].

The Pharmacy Health Information Technology Collaborative (Pharmacy HIT) was created in 2010 by nine national professional organizations, comprising practice, education, and accreditation as well as system vendors, e-prescribing networks, and standards-development groups, to facilitate integration of pharmacy practice and medication management into an evolving health information technology (HIT) infrastructure [5]. A strategic plan was formulated in 2011 and recently updated through 2017. Of the 10 goals recommended in the Pharmacy HIT’s roadmap, six of these have direct importance for clinical pharmacy practice related to paediatrics patients [5]. These include: (1) ensuring that HIT supports pharmacists in health care service delivery; (2) achieving integration of clinical data with electronic prescription (eRx) information; (3) ensuring that HIT infrastructure includes and supports MTM services; (4) integrating pharmacist-delivered immunizations into electronic health records (EHR); (5) achieving recognition of pharmacists as meaningful users of EHR quality measures; and (6) achieving integration of pharmacies and pharmacists into health information exchanges [5]. Within each of these goals, Pharmacy HIT prepared a list of strategies to assist with the optimization of pharmacist involvement in HIT-related functions and processes (Table 1). While specific patient parameters, such as age, disease, or medication management issues such as scientifically-validated CNP formulations are not mentioned, the initiative provides a comprehensive framework for concerted action on pharmacy-related HIT issues and concerns.

**Table 1:** Pharmacy HIT Collaborative 2017 Goals and Key Strategies that impact Pharmacy Paediatrics [5].

2017 Pharmacy HIT Goals	Key Strategies Related to Pharmacy Paediatrics
Goal 1: Ensure that HIT supports pharmacists in health care service delivery	Develop white papers describing the appropriate flow of critical electronic information among health care providers, including pharmacists, that protects patient privacy while providing medical information needed for decision making for optimal therapy.
Goal 2: Achieve integration of clinical data with electronic prescription	Engage and participate in standards-setting organizations, task forces, and work groups to improve electronic exchanges related to e-prescribing.
Goal 4: Ensure that HIT infrastructure includes and supports MTM services	Work with organizations defining the pharmacist’s MTM role in HIT, such as pharmacy associations, PSTAC, MTM intermediaries, and NCPDP, to ensure that MTM principles and guidelines defined by pharmacists are incorporated into the national HIT infrastructure.
Goal 5: Integrate pharmacist-delivered immunizations into EHR	Enhance the ability of pharmacists to electronically document, share, and evaluate patient immunization therapy.
Goal 6: Achieve recognition of pharmacists as meaningful users of EHR quality measures	Ensure that pharmacists are involved in the determination and adoption of the meaningful use of the EHR quality measures pertaining to medications and medication-related activities
Goal 9: Achieve integration of pharmacies and pharmacists into health information exchanges	Work with policymakers, including state Medicaid agencies, ONC, CMS, HHS, and other members of the health care industry, to promote the importance of pharmacist participation in HIEs

The purpose of this paper is to identify key positions within the health information technology infrastructure where an electronic repository for medication management related to paediatric compounded non-sterile products (pCNP) and information exchange would be inserted optimally to facilitate and maintain transmission of e-prescriptions (eRx) from initiation to fulfillment. Further, the paper will propose key placement requirements for such a repository to provide for interoperability of electronic medication management systems to minimize untoward disruptions across the continuum of care.

## 2. National Electronic Health Information Infrastructure (HIT)

**Figure 1:**
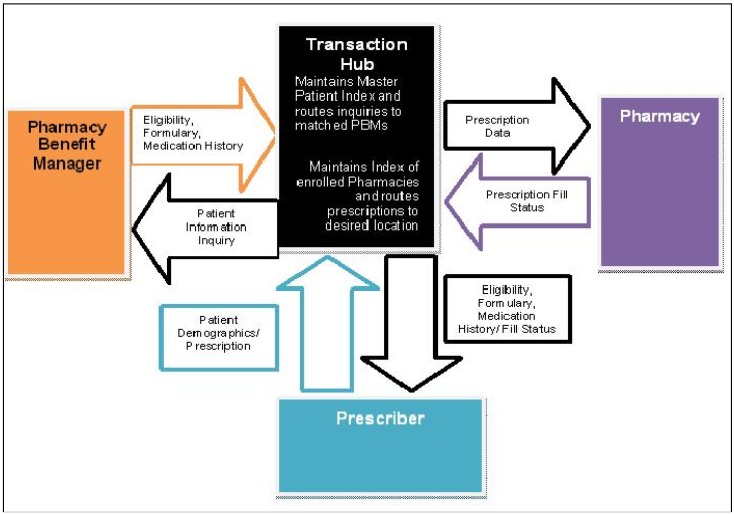
High-level dataflow diagram outlining the roles and processes involved in eRx [9].

Production of the informational infrastructure for health care was stimulated in 1994 through focused program competition sponsored by the US National Institute for Standards and Technology with an estimated budget of $185 million USD over five years [6]. The purpose of this private-public partnership was to enable enterprise-wide integration of information among all sectors of healthcare [7]. Components of this infrastructure, designed based on user requirements, included: (1) reliable storage and retrieval of complex medical information for varied applications; (2) real-time, data-driven medical decisions; (3) real-time data entry by mobile medical personnel; (4) real-time global transport of complex medical records with accuracy, speed, and security; and (5) computer-based medical training, diagnostic, and reference tools. Many of these system components have been addressed in subsequent work that developed reliable user-interfaces and efficiency-enhancement technologies such as user information repositories [8]. Recipients of major funding awards related to HIT infrastructure included C. Everett Koop Institute ($45 M), Health Data Sciences Corporation ($22.5 M), and First Data Health Systems Corporation ($14 M). As a result over the last 20 years, the Office of the National Coordinator for Health Information Technology (ONC) reported dramatic progress in connectivity of electronic health information and interoperability of information technologies [7]. However, ONC noted recently that eRx functionality in medication use and management systems “has yet to be fully realized” [8]. A high-level illustration of eRx system architecture is found in Figure 1.

## 3. Pharmacy-Related HIT across the Continuum of Care for Paediatric Patients

### 3.1. Initiation and Generation of eRx from the Provider

Within the current electronic medication management system configuration, a number of special circumstances unique to children impact the safety and effectiveness of their pharmacotherapy [10], and have been noted elsewhere in this Special Edition [11,12]. The point of entry for eRx is in the provider’s office or in an institution such as hospitals. In addition to fixed location-based transmission, Ventola reviewed the multitude of medical applications housed on mobile devices [13]. Many providers have eRx-generation software as extensions of a stationary location, such as their offices, to enable on-the-go transmission from a tablet or smart phone. Prevalent physician themes regarding mobile device use for eRx transmission uncovered by Agarwal and colleagues in a recent qualitative study were: (1) eRx as an efficiency and effectiveness enhancing tool; (2) eRx as the harbinger of new practices; (3) eRx as core to the clinical workflow; (4) eRx as an administrative tool; (5) eRx: the artifact; (6) eRx as a necessary evil; and (7) eRx as an unwelcome disruption [14]. Regardless of theme, it is apparent that an overwhelming number of providers no longer hand-write most of their prescriptions. However, in the context of paediatric drug therapy, most eRx for children continue to be generated free-text without the benefit of allergy screening, dose-range checking, therapeutic duplication, or posology/nosology clinical decision support, let alone clinical monitoring and following requirements necessary for safe and effective medication management [15].

In the institutional environment, a 2013 US national survey of hospital pharmacies found that almost 60% of hospitals had CPOE systems with the capacity to transmit eRx to outpatient pharmacies [16]. In these systems, the hospital’s formulary is used often as the database for creation of the outpatient eRx order. The formulary listing is codified with site-specific formulation identifiers that are not shared with or recognized by external systems. Thus, when eRx is generated in the hospital setting, transmission is impeded by the lack of uniform product codes and descriptors. As Gracy and others have noted, “Available EHR systems generally do not adequately reflect the needs of children and pediatric care providers” [17]. This is especially true of prescription-generation functions within these systems, and there is little appreciation of the complexity of complete eRx creation and transmission for children’s therapies [18]. In a recent study, 13.6% of all e-prescriptions generated and transmitted from the emergency department (ED) of a major US children’s hospital to community pharmacies during a one-year period resulted in a significant error that required the pharmacy to contact the ED for clarification [19].

### 3.2. Transmission from Provider to Intermediary

When the provider sends eRx electronically, the formatted message is transmitted to an intermediary or transaction hub. This intermediary serves as an emulator for individual authorizations and sharing of patient-specific communication related to prescription therapies. Almost one-half of all prescriptions in a recent study sent electronically contained free-text format, often with duplicate or missing information contained within various code fields, creating opportunities for error and rework [20]. In another study, Zhou and colleagues found the proportion of free-text prescription entries for hypoglycemic agents within a paediatric clinic to be 14.9% [21]. Moreover, the provider often does not have the advantage of previewing the eRx before it is transmitted [22]. One major EHR software vendor recommends that paediatric providers generate hard-copy prescriptions for children as a workaround due to the lack of an effective means for electronic processing of pCNPs [23].

### 3.3. Emulation at the Intermediary

**Figure 2:**
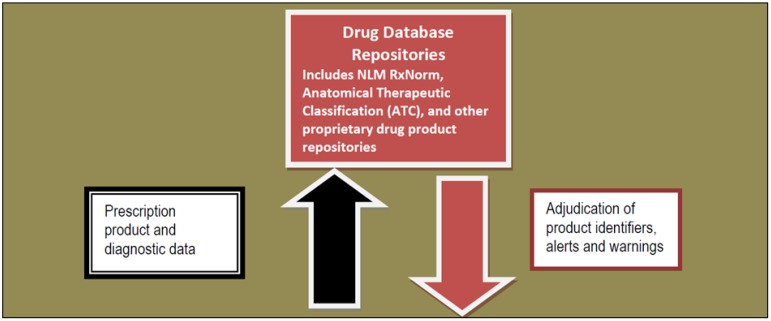
Positioning Drug Databases within the eRx architecture.

Once received in the transaction hub, the intermediary formats eRx information and data elements using National Council of Prescription Drug Program (NCPDP) SCRIPT Standards [24]. These standards include the data structure for: (1) new prescriptions; (2) changes to a new prescription; (3) cancellation of prescription; (4) refills/renewals/resupplies; (5) fill status notification; (6) medication history exchange; (7) drug administration exchange in long term care; (8) prescriber-reported sample distribution; and (9) query functions for new prescriptions [20]. NCPDP’s data dictionary contains actual data field descriptors, character field length, formats, comments, and usage instructions [24]. In addition, formulary, benefit, and prior authorization standards are included to aid in the selection of the most appropriate medication for the patient. As noted in the draft 2016 Interoperability Standards Advisory, the HIT industry is moving toward use of RxNorm for current prescribable content [25]. Moreover, Bodenreider and Rodriguez demonstrated the feasibility of using Anatomical Therapeutic Classification (ATC) in conjunction with RxNorm for analyzing eRx datasets for assessment of prescribed daily doses [26]. Taken together, combining these systems may provide an opportunity to identify and categorize off-label medication use. See Figure 2 for the relational position of RxNorm and other drug databases.

### 3.4. Order Fulfillment at Community Pharmacy, Sub-Acute Hospital, Home Care Pharmacy, or Pharmacy Benefit Manager (PBM)

Upon receiving these NCPDP-structured eRx transmissions, often the community pharmacy, sub-acute hospital, home care pharmacy, or PBM needs to re-enter eRx in a format compatible with their pharmacy information systems. The dispensing pharmacy may have received a dose expressed in volume (*i.e*., mLs) without a corresponding solution concentration, creating re-work and the need to contact the prescriber for desired liquid strength. Once clarified, the pharmacy may need to re-enter the medication as a free-text due to the lack of standardized product listing or system allergy and alert files linked to the actual drug product.

## 4. Where in the Infrastructure could a pCNP Repository Be Inserted?

In an ideal world, changes to product listing data in the electronic environment would be subjected to simulation across the continuum of care as an ongoing quality assurance process. The Rand Corporation study group conducted a live pilot test of RxNorm in ambulatory care using five physician office practices, two community pharmacies, and one mail-order pharmacy as test sites [27]. While beta-testing of this nature within the medication use system is not possible at this time, ideally, the placement of a standardized, evidence-based pCNPeRx repository within the HIT infrastructure might have the following requirements:

- Ease of integration into RxNorm-formatted drug coding structures;
- Ease of maintenance and updating for new and modified extemporaneous pCNP formulations;
- Ability to emulate compatible and complete eRx transmission from initiation to fulfillment;
- Ability to facilitate bi-directional integration of clinical use data related to a minimum data set of patient assessment factors such as indication or purpose, dose-response relationships, and outcomes;
- Capability to create medication lists that would aid medication reconciliation across the continuum of care; and
- Ease of third-party adjudication for pCNP preparation and dispensing.

**Figure 3:**
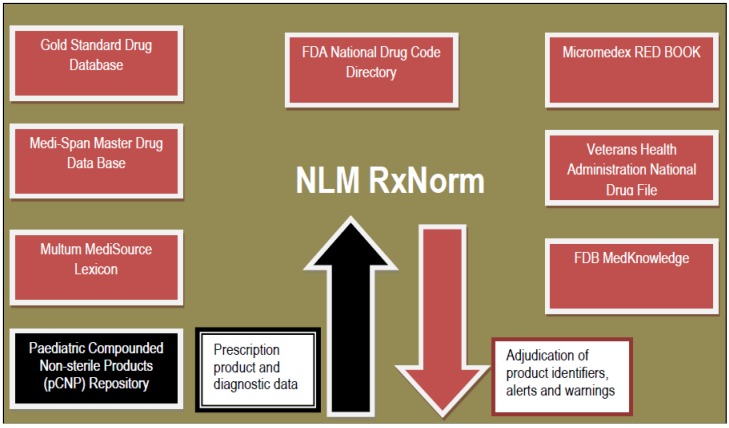
Drug Databases that Articulate with NLM RxNorm with pCNP positioning (proposed) [29].

In early 2015, USP published a compendium of compounded products that have scientific evidence for formulation stability and potential applicability in paediatric patient care [28]. This compendium includes the descriptor, “compounded oral suspensions”, for many of its listed CNPs, creating a new official dosage form description and providing a certifiable extemporaneous process for the preparation of listed products. Regardless of the electronic repository’s ultimate owner and with the above criteria in mind, a standardized, evidence-based formulation repository could be housed best within the purview of a centralized repository structure, like RxNorm or a proprietary database listed in Figure 3. Placing an electronic dataset for pCNP formulations near the transaction hub and employing RxNorm product nomenclature and identification schema would increase the likelihood of accurate and complete eRx transmission for pCNP formulations.

## 5. Conclusions

The optimal placement of a repository for paediatric CNP formulations within the health information infrastructure may be within the same position as other drug databases, although there is a corollary need to populate end-user medication listings and data structures. In order for information to propagate from initiation and through every system node to order fulfillment, the electronic identification of products as well as a standardized set of clinical descriptors, many of which are unique to paediatric patients, must be recognized and formatted uniformly. Moreover, the requirement for bi-directional information sharing is a key concept in improving the safety and effectiveness of pharmacotherapy for children. Ultimately, access to and safety of vitally-needed CNP pharmacotherapies for children would be enhanced and assured within the electronic environment across the continuum of care.
